# SDS Electrophoresis on Gradient Polyacrylamide Gels as a Semiquantitative Tool for the Evaluation of Proteinuria

**DOI:** 10.3390/diagnostics13091513

**Published:** 2023-04-23

**Authors:** Paulina Mazur, Paulina Dumnicka, Joanna Tisończyk, Anna Ząbek-Adamska, Ryszard Drożdż

**Affiliations:** 1 Department of Medical Diagnostics, Faculty of Pharmacy, Jagiellonian University Medical College, 30-688 Kraków, Poland; 2 Department of Diagnostics, University Hospital in Kraków, 30-688 Kraków, Poland

**Keywords:** kidney disease, glomerular proteinuria, tubular proteinuria, overload proteinuria, sodium dodecyl sulphate–polyacrylamide gel electrophoresis

## Abstract

Proteinuria is an important sign of kidney diseases. Different protein patterns in urine associated with glomerular, tubular and overload proteinuria may be differentiated using the immunochemical detection of indicator proteins or via urinary proteins electrophoresis. Our aim was to characterize sodium dodecyl sulphate–polyacrylamide gel electrophoresis (SDS-PAGE) using commercially available 4–20% gradient gels as a method to detect and differentiate proteinuria. Our laboratory-based study used excess urine samples collected for routine diagnostic purposes from adult patients of a tertiary-care hospital, including patients with albumin/creatinine < 30 mg/g and patients with dipstick proteinuria. The limit of albumin detection was estimated to be 3 mg/L. In 93 samples with albumin/creatinine < 30 mg/g, an albumin fraction was detected in 87% of samples with a minimum albumin concentration of 2.11 mg/L. The separation of 300 urine samples of patients with proteinuria revealed distinct protein patterns differentiated using the molecular weights of the detected proteins: glomerular (albumin and higher molecular weights) and two types of tubular proteinuria (“upper” ≥20 kDa and “lower” with lower molecular weights). These patterns were associated with different values of the glomerular filtration rate (median 66, 71 and 31 mL/min/1.72 m^2^, respectively, *p* = 0.004) and different proportions of multiple myeloma and nephrological diagnoses. As confirmed using tandem mass spectrometry and western blot, the SDS-PAGE protein fractions contained indicator proteins including immunoglobulin G, transferrin (glomerular proteinuria), α1-microglobulin, retinol-binding protein, neutrophil gelatinase-associated lipocalin, cystatin C, and β2-microglobulin (tubular), immunoglobulin light chain, myoglobin, and lysozyme (overflow). SDS-PAGE separation of urine proteins on commercially available 4–20% gradient gels is a reliable technique to diagnose proteinuria and differentiate between its main clinically relevant types.

## 1. Introduction

Chronic kidney disease (CKD) is a worldwide public health problem. The prevalence of CKD and its most advanced stage, end-stage renal disease (ESRD), continues to increase [1]. Irrespective of the initial cause, CKD is a progressive disease. Late diagnosis is associated with reduced possibilities for nephroprotective treatment and poor prognosis [2].

Urine is an important source of clinical biomarkers of kidney disease. Urine samples can be obtained non-invasively, repeatedly, and in sufficient amounts. Elevated protein concentrations in urine should always be considered an alarming signal, indicating possible kidney disease [3]. The urinary excretion of more than 150 mg total protein per day is considered increased proteinuria. Current Kidney Disease–Improving Global Outcomes (KDIGO) CKD guidelines recommend the measurements of albumin in urine and define urinary albumin excretion of 30–300 mg per day as moderately elevated albuminuria (previously called microalbuminuria) and ≥300 mg per day as significantly elevated or severe albuminuria [4]. Proteinuria/albuminuria is not only a sign of renal disease but also an independent risk factor for the progression of kidney disease and mortality [4,5]. In clinical studies, the extent of proteinuria correlates with a faster decline of the glomerular filtration rate (GFR) in patients with CKD. Therefore, the strict monitoring of proteinuria helps in the optimal management of patients and the use of nephroprotective therapies aimed at lowering proteinuria [6,7,8,9]. Increased proteinuria has also been associated with adverse outcomes in other clinical settings. In a large cohort of general surgery patients, increased levels of preoperative proteinuria predicted post-surgery acute kidney injury and 30-day readmission independent of the GFR [10]. In patients with kidney cancer, proteinuria present before partial or radical nephrectomy was significantly associated with adverse renal outcomes and shorter overall survival [11].

Proteinuria can be a consequence of a wide variety of both systemic and renal disorders [12]. The etiology affects the composition of the urine proteome. The determination of the protein composition of urine may help to noninvasively diagnose renal lesions and evaluate therapeutic interventions [13]. Several main types of proteinuria have been distinguished based on the pathomechanism. The most common cause of proteinuria of renal origin is the damage to the glomerular filtration barrier (glomerular proteinuria), and less frequently to the renal tubular cells (tubular proteinuria). However, both the glomeruli and the tubules may be involved to various extents in a single patient. Moreover, overload proteinuria may result from increased plasma concentrations of low molecular weight proteins that are filtered in excess through the glomeruli; extrarenal proteinuria is related to pathological processes involving the lower urinary tract [14,15]. In this complex situation, it is obvious that a single marker such as urinary albumin cannot provide a clear indication on the cause of kidney disease [16,17].

Nonetheless, an early and accurate recognition of the presence of proteinuria and the underlying cause is essential to provide appropriate patient care. In current clinical practice, the type of proteinuria may be assessed based on immunochemical measurements of selected “indicator” proteins: low molecular weight (LMW) proteins (i.e., with molecular mass lower than albumin) such as retinol-binding protein, β_2_-microglobulin, α_1_-microglobulin and cystatin C for tubular proteinuria; albumin and transferrin for glomerular proteinuria; and free light chains of immunoglobulin, myoglobin or lysozyme for overload proteinuria [18,19,20,21,22]. However, since immunochemical assays are relatively expensive, this diagnostic approach requires the initial diagnostic hypothesis: the initial misconception may substantially extend the entire diagnostic process.

One of the possible technical solutions is the electrophoretic separation of urine proteins. Increased permeability of the glomerular capillary wall and increased trans-glomerular passage of middle molecular weight proteins or impaired reabsorption of low molecular weight proteins by the epithelial cells of the proximal tubules result in distinct urine protein profiles, which may be differentiated on the basis of the molecular weight [23]. Sodium dodecyl sulfate–polyacrylamide gel electrophoresis (SDS-PAGE) is a simple, inexpensive method for the separation of proteins according to their molecular weight [24]. In medical laboratory diagnostics, SDS-PAGE is considered a reference method for distinguishing distinct types of proteinuria (glomerular, tubular, overload, and extrarenal) that are associated with distinct patterns of the urinary protein fractions and their relative proportions. The intensity of the obtained protein bands provides additional information on the concentrations of a specific proteins in the urine [25,26]. Nevertheless, SDS-PAGE is presently seldom used in routine medical laboratories due to the time-consuming process, the need for manual work, and the belief of low analytical sensitivity. However, the use of commercially available polyacrylamide gels and reagents significantly accelerates the diagnostic process and enables semiquantitative assessment of the concentrations of selected proteins present in urine.

The aim of the study was to characterize and evaluate the SDS-PAGE using commercially available polyacrylamide gradient gels as a method for the detection and differentiation of the types of proteinuria in human samples.

## 2. Materials and Methods

### 2.1. Study Design and Samples

This was a medical laboratory-based cross-sectional observational study, utilizing urine samples collected from adult patients treated in a tertiary-care center, the University Hospital in Kraków, Poland, in the years 2018–2019. For the purpose of the study, the excess samples remaining after routine laboratory examinations were chosen based on the results of the laboratory tests ordered and performed during the routine diagnostic process (urinalysis, urine total protein or albumin concentration and albumin/creatinine ratio, serum creatinine, and estimated glomerular filtration rate—eGRF). After completing the routine laboratory tests, the excess urine samples were centrifuged (400× *g*; 5 min) and the supernatant was aliquoted, frozen, and stored at −20 °C until use.

We included three groups of samples:

- A total of 93 samples from patients with normal urinalysis results and urine albumin/creatinine ratio (ACR) < 30 mg/g were used to verify the limit of detection of the studied method and to observe a normal SDS-PAGE protein pattern;
- In total, 300 samples from patients with proteinuria of varying severity, detectable in routine urinalysis, and with a broad range of eGFR, were used to study glomerular and tubular patterns of proteinuria; from this group, selected samples were also used to identify proteins present in SDS-PAGE fractions;
- Ten samples from patients with hemoglobinuria detected with urinalysis were used to study the SDS-PAGE protein pattern associated with hemoglobinuria.

The patient data collected for the purpose of the study included age, sex, eGFR, and the information about the abnormal results of urinalysis (proteinuria or hemoglobinuria). In case of 300 samples with proteinuria, the available International Statistical Classification of Diseases and Related Health Problems 10th Revision (ICD-10) codes accompanying the orders for routine laboratory tests were retrieved from the laboratory information system.

The samples and patients’ data were anonymized while collected for the study purposes and the data enabling the identification of patients were not recorded during the study.

### 2.2. Ethical Statement

The study protocol was approved by the Bioethical Committee of the Jagiellonian University no. 122.6120.58.2017 issued on 30 March 2017. The requirement for obtaining patients’ informed consent for the study was waived.

### 2.3. SDS-PAGE

Sodium dodecyl sulphate (SDS) electrophoresis was conducted on 12.5% self-made polyacrylamide gels and on commercially available gradient (4–20%) polyacrylamide gels, as specified in Results.

Self-made polyacrylamide gels were used with self-prepared sample buffer (4 mL of 0.1% bromophenol blue solution, 4 mL of 40% sucrose solution, and 8 mL of 20% SDS solution) and electrode buffer (30 g of Trizma base, 10 g of SDS, and 144 g of aminoacetic acid in 1 L of distilled water, pH 8.3). SDS, acrylamide, bromophenol blue, Trizma base, aminoacetic acid, and TEMED from Sigma–Aldrich, and ammonium persulfate (APS), hydrochloric acid, butanol, methanol, Coomassie Brilliant Blue G 250, perchloric acid, citric acid and sucrose from Chempur, Poland were used to prepare the self-made gels and buffers. The detailed procedures are presented in Supplementary File 1.

Commercially available gradient polyacrylamide gels, the TruPAGE Precast Gels 4–20%, were obtained from Sigma–Aldrich (lot: 161017100). Gradient gels were used with TruPAGE LDS Sample Buffer (Sigma–Aldrich, St. Louis, MO, USA, lot: 15113002) and TruPAGE TEA—Tricine SDS Running Buffer (Sigma–Aldrich, lot: 141103003), according to the instructions of the manufacturer. As a molecular weight standard, Color Prestained Protein Standard (New England BioLabs, Ipswich, MA, USA, lot: 0101703 and 10083356) was used according to the instruction of the manufacturer. Urine samples of patients with monoclonal gammopathy containing albumin and free immunoglobulin light chains (dimer and monomer) were used as internal molecular weight standards (in some experiments, lysozyme and myoglobin were added to the samples).

Urine samples, thawed before use, were mixed with TruPAGE LDS Sample Buffer (Sigma–Aldrich), or a self-prepared sample buffer in 1:1 ratio and 20 µL of the mixture was added per well. The electrophoresis was conducted in the Mini-Protean MP-300 V System (BIO-RAD, Hercules, CA, USA) for 45 min, at 180 V. After the electrophoresis was completed, gels were placed in the stain solution (0.4 g of Coomassie Brilliant Blue G 250 was mixed with 500 mL of deionized water and 25 mL of perchloric acid) for 30 min. Destaining (in 0.1% citric acid) was conducted for 2–3 days, until the stain solution was completely removed. Gels were scanned using Epson Perfection V500 Scanner. Densitometry of the gels were performed using DensyGraf software designed by the author (R.D.).

### 2.4. Western Blot

A wet transfer of proteins was performed from polyacrylamide gradient gels to a nitrocellulose membrane (BIO-RAD). The transfer was conducted in Mini-Protean MP-300 V System (BIO-RAD) in a basic buffer (3.03 g of Trizma base, 14.4 g of aminoacetic acid and 200 mL of 20% methanol, adjusted to a volume of 1 L with distilled water, pH 8.3) for 1 h, at 100 V.

The identification of proteins on the nitrocellulose membrane was performed using rabbit primary antibodies: anti-uromodulin (Sigma–Aldrich, lot: R39634; dilution 1:250), anti-cystatin C (Merck, lot: 2738995; dilution 1:1000), anti-α1-microglobulin (Bioassay Technology Laboratory, lot: BT-AP06932; dilution 1:1000), and enzymatically labelled (horseradish peroxidase) goat anti-rabbit secondary antibody (Merck, lot: 2857930; dilution 1:1000). All antibodies were used according to the instructions of the manufacturers. As a substrate for horseradish peroxidase, 1% 3-amino-9-ethylcarbazole (Sigma–Aldrich) was used. Membranes were scanned using Epson Perfection V500 Scanner.

### 2.5. Liquid Chromatography and Tandem Mass Spectrometry (LC-MS/MS)

The composition of protein fractions detected in a selected urine sample was studied using liquid chromatography–tandem mass spectrometry. The urine proteins were separated with SDS-PAGE using a 4–20% gradient gel (Sigma–Aldrich) stained with Coomassie Brilliant Blue G 250 as described above. All visible bands were excised with a scalpel and each placed in a separate vial.

Sample preparation as well as LC-MS/MS measurements for protein identification from gel bands were performed as described in Pabis et al. [27], with minor modifications. Protein digestion was performed with trypsin (200 ng per sample) suspended in 25 mM ammonium bicarbonate. Samples were incubated with enzyme overnight at 37 °C. Peptide separation during the LC-MS/MS analysis was performed at a flow rate of 250 nL/min. The collected LC-MS/MS data were processed using the Proteome Discoverer software (v. 2.1; Thermo Scientific, Waltham, MA, USA) and searched using an in-house MASCOT server (v. 2.5.1; Matrix Science, London, UK) against SwissProt database restricted to Homo sapiens taxonomy (20 387 sequences). The database search was performed assuming carbamidomethylation of all cysteines as well as the possibility of methionine oxidation and protein N-term acetylation. Tryptic peptides with up to 1 missed cleavage allowed were considered during the search. Peptide identification was done with precursor mass tolerance of 10 ppm and fragment mass tolerance of 20 mmu. The false discovery rate (FDR) threshold for protein identification was set to 1%.

The lists of proteins obtained on LC-MS/MS analysis were analyzed as follows: first, for each protein fraction analyzed, we selected the LC-MS/MS detected proteins with molecular weights similar to the molecular weight of the protein fraction; furthermore, the list of LC-MS/MS detected proteins in each fraction was restricted to those with the highest number of identified peptides, preferably above 10. The aim of the analysis was to obtain a short list of proteins abundant in each SDS-PAGE fraction.

### 2.6. Albumin and Immunoglobulin Light Chain Concentration in Urine

The concentration of albumin and immunoglobulin light chains in urine was determined using the immunonephelometric method on the Siemens BN II analyzer.

### 2.7. Statistical Analysis

Categorical data were presented as the number (*n*) of cases or samples and percentage of the respective group. Quantitative data were summarized using the median, lower quartile (Q1) and upper quartile (Q3), or range, as specified in the Results. All studied quantitative variables were non-normally distributed (as verified using the Shapiro–Wilk test). The contingency tables were analyzed with the Pearson χ^2^ test or Fisher exact test (expected counts < 5). Non-parametric tests (Mann–Whitney or Kruskal–Wallis, according to the number of groups) were used to study differences between the groups considering quantitative data. Linear and polynomial regression was used to fit curves to the experimental data. The tests were two-tailed and the results were considered statistically significant at *p* < 0.05. Statistica 13.3 software (Tibco, Tulsa, OK, USA) was used for computations.

## 3. Results

### 3.1. Comparison of Urine Protein Separation Using Commercially Available 4–20% Gradient Polyacrylamide Gels with Self-Cast Gels

**Figure 1:**
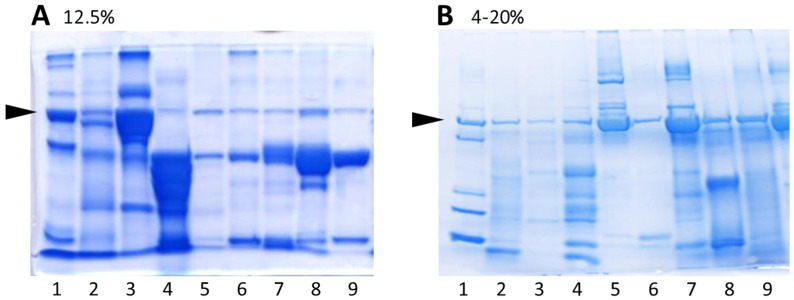
SDS-PAGE separations of nine urine samples on self-cast polyacrylamide gel with different acrylamide concentrations: 12.5% (**A**) and on commercially available gradient gel of 4–20% (**B**). The same nine samples were used (1–9). The arrowheads indicate the fraction containing albumin.

To compare SDS-PAGE using self-cast and commercially available gels and buffers, urine proteins were separated on 12.5% polyacrylamide gels as well as on commercially available gradient gels. Figure 1 shows electrophoretic separations of the same nine urine samples using polyacrylamide gels with different acrylamide percentage. Sample number 1 was an internal standard (a urine sample containing albumin and immunoglobulin light chains in the form of both dimers and monomers with added myoglobin and lysozyme). The use of 4–20% gradient gels enabled the electrophoretic separation of proteins with a wide range of molecular weights; therefore, gradient gels were used for further studies.

### 3.2. Analytical Performance of Urine Protein Separation Using SDS-PAGE 

**Figure 2:**
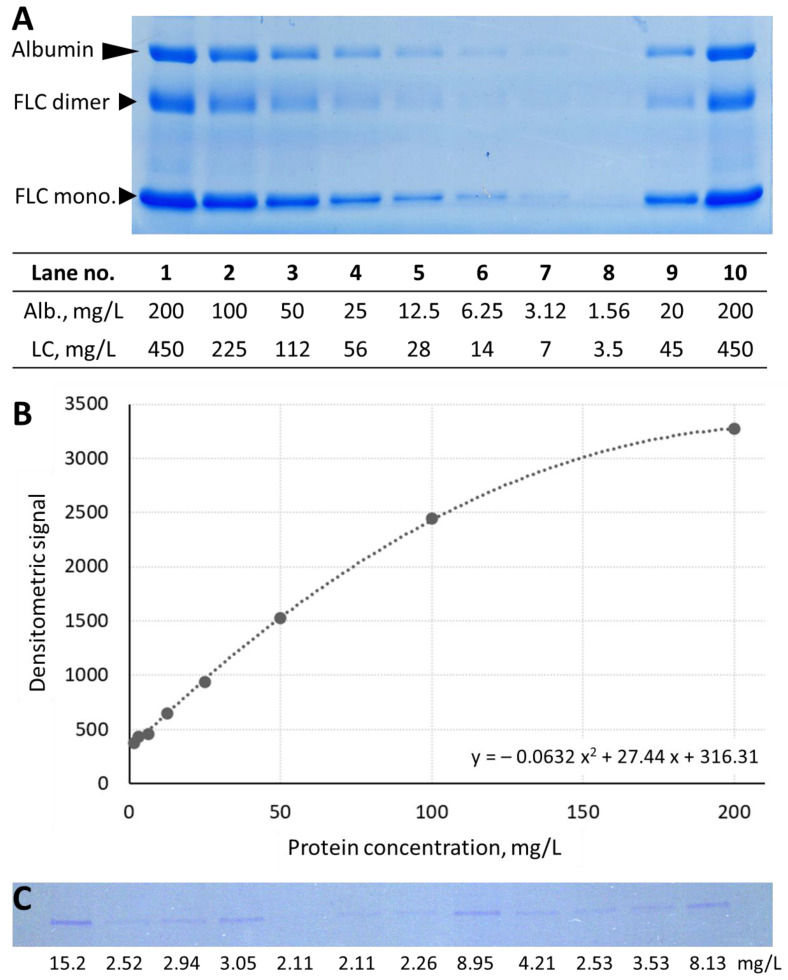
Panel (**A**) SDS-PAGE separations of urine samples with albumin (Alb.) concentrations of 1.56–200 mg/L and immunoglobulin light chain (LC) concentrations of 3.5–400 mg/L. The table below the scan of the gel shows the concentrations of albumin and LC in each lane. FLC dimer–immunoglobulin free light chain dimer, FLC mono–immunoglobulin free light chain monomer. Panel (**B**) The association between the protein (albumin) concentration and the densitometric signal. Panel (**C**) The albumin band observed in SDS-PAGE in urine samples of patients with normal urinalysis; albumin concentration in each sample is provided below the gel’s scan.

To study the detection limit of urinary protein in SDS-PAGE analysis, a series of dilutions of urine sample containing albumin and monoclonal immunoglobulin free light chain (FLC) was separated using SDS-PAGE and stained with Coomassie brilliant blue. The immunochemically measured concentration of albumin in the original sample was equal to 200 mg/L. The dilutions were prepared to obtain samples with decreasing albumin concentration of 1.56 mg/L. The electrophoretic separation of the prepared urine samples (Figure 2) allowed the visualization of the albumin band at the concentration of 3.12 mg/L. Light chain concentrations in the dilutions were between 7 and 450 mg/L. As shown in Figure 2A, FLC dimeric and monomeric forms were observed.

To confirm the detection limit of the method, samples from 93 patients (49 women, 44 men; age between 22 and 86 years, median 59 years) with normal urinalysis results and ACR < 30 mg/g were separated on 4–20% polyacrylamide gradient gels. Urine albumin concentrations in the samples were between 2.11 and 31.2 mg/L (median concentration 4.17 mg/L). An albumin band was detected in 81 (87%) patients with a minimum albumin concentration of 2.11 mg/L. For comparison, in 12 samples with non-detectable albumin band, urine albumin concentrations were between 2.11 and 4.73 (median 2.11) mg/L. An albumin band in SDS-PAGE was detected in 19 (63%) of 30 urine samples with albumin concentrations below 3 mg/L and in 62 (98%) of 63 samples with albumin concentrations ≥ 3 mg/L. A representative scan of albumin bands in patient samples is presented in Figure 2C.

**Figure 3:**
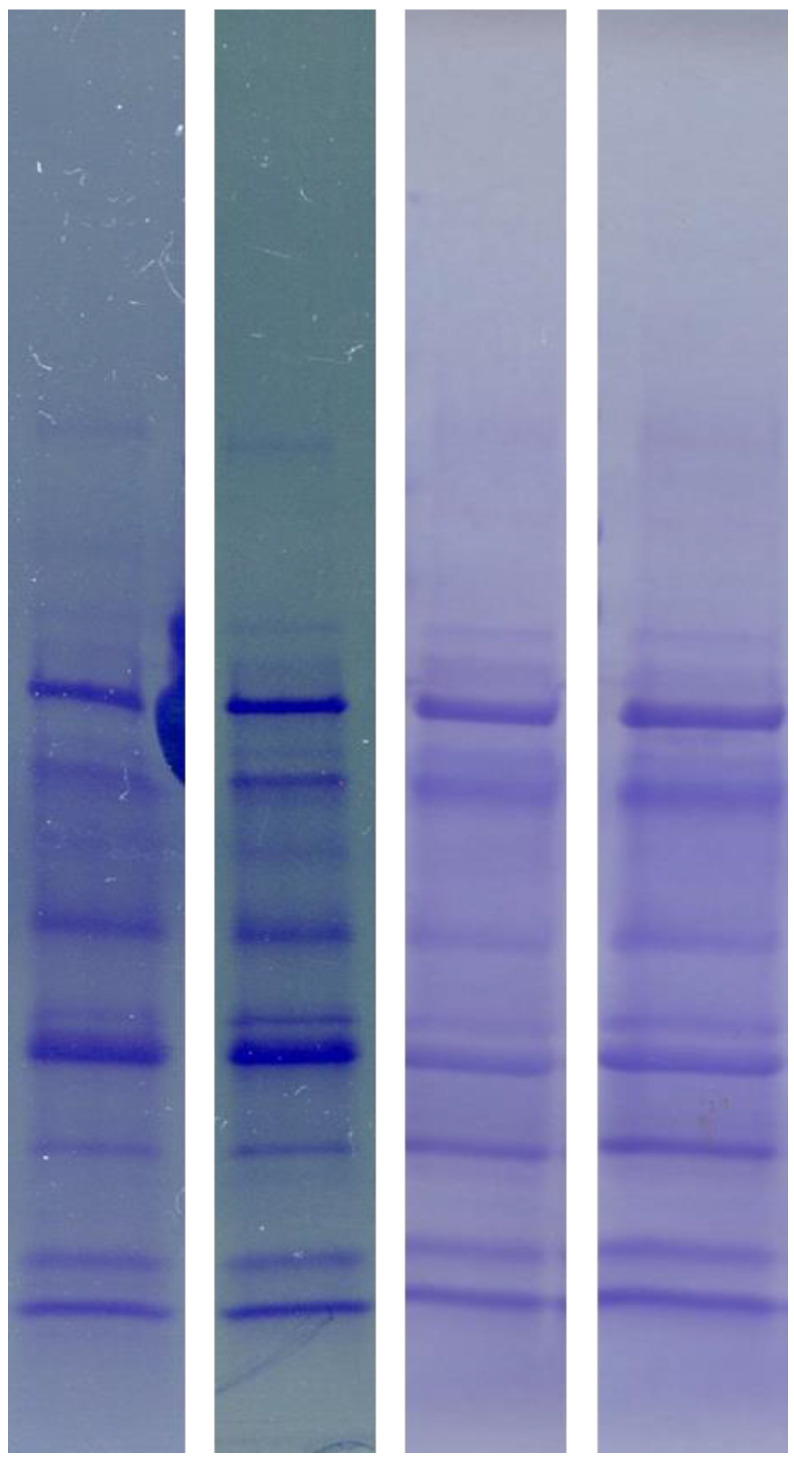
Urine samples from the same patient separated with SDS-PAGE on four different gels.

As shown in Figure 3, SDS-PAGE allowed for repeatable results. Both in the case of electrophoretic separations of the same urine sample on different gels, and for samples obtained from one patient at different times, comparable urine protein profiles were obtained.

### 3.3. Identification of Urine Protein Fractions Obtained with SDS-PAGE Separation Using 4–20% Gradient Gels

**Figure 4:**
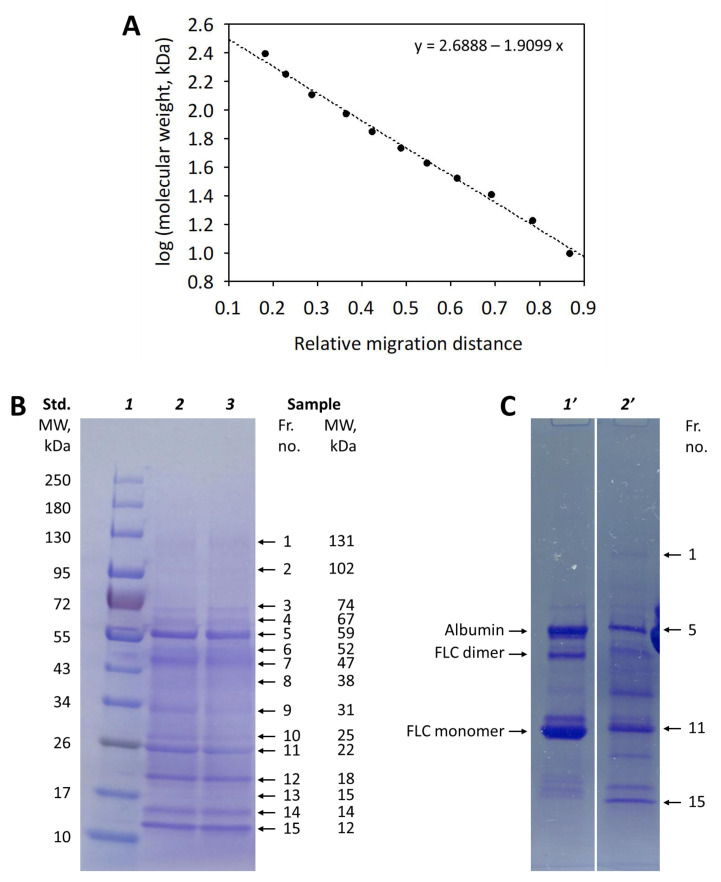
Panel (**A**) Molecular weight calibration curve based on SDS-PAGE separation of molecular weight standard (New England BioLabs) on 4–20% polyacrylamide gradient gel; Panel (**B**) Electrophoretic separation of molecular weight standard (line *1*, used to produce the standard curve) and a patient’s urine sample (in duplicate, lines *2* and *3*). “Std. MW” indicates the molecular weights of proteins included in the standard. The 15 protein bands (fractions) were identified and numbered (Fr. no.) in the sample, and their molecular weights (MW) were estimated based on the standard curve. Panel (**C**) The same patient’s urine sample (line *2′*) separated on 4–20% polyacrylamide gradient gel compared with known urine samples containing albumin and free immunoglobulin light chain (FLC) dimers and monomers. Protein fraction 5 may be identified as albumin on the basis of the migration distance.

To determine approximate molecular weights of the SDS-PAGE separated proteins, a molecular weight calibration curve was prepared via separation of a commercially available molecular weight standard containing eleven different molecular weight markers ranging from 10 to 250 kDa. Based on the calibration curve, the molecular weights of urinary proteins were estimated (Figure 4).

Further analysis of protein fractions separated using SDS-PAGE on 4–20% gradient gels utilized LC-MS/MS of peptide mixtures obtained via excision of single bands from the polyacrylamide gel followed by reduction and trypsin digestion of proteins. For the analysis, a sample containing multiple low molecular proteins (i.e., with molecular weight lower than the molecular weight of albumin) was chosen (shown in Figure 4B, lines *1* and *2*). All the 15 protein fractions that were visualized using Coomassie brilliant blue staining were excised and used for the identification of proteins using tandem mass spectrometry. The results of LC-MS/MS in the form of a full list of proteins identified in each fraction analysis are provided as Supplementary File 2. The proteins identified based on the highest numbers of peptides (chosen as described in the Methods) are presented in Table 1.

In the next step, western blot analysis was performed to confirm the identification of selected urinary proteins. Following SDS-PAGE separation of 12 urine samples with tubular and mixed glomerular-tubular proteinuria, the proteins were transferred to a nitrocellulose membrane and identified using specific antibodies. The western blots confirmed the presence of uromodulin, α_1_-microglobulin, and cystatin C in urine samples (Supplementary Figure S1).

### 3.4. Urine Protein Patterns Obtained Using SDS-PAGE on 4–20% Gradient Gels in Patients’ Samples

The separation of 300 urine samples of patients with proteinuria (126 women, 174 men; age between 18 and 91 years, median 64 years) using the described SDS-PAGE method (4–20% gradient gels) allowed the observation of distinct protein patterns characteristic for the main clinically distinguished types of proteinuria: the glomerular and the tubular proteinuria. We observed pure glomerular patterns characterized by the presence of albumin and the proteins of higher molecular weights in 96 samples (32%). In 204 samples (68%), low molecular weight proteins were present, characterizing tubular proteinuria with or without the glomerular component. Sex distribution was similar in the two subgroups (*p* = 0.7); however, the patients with pure glomerular proteinuria were younger (median 61 versus 66 years, *p* = 0.003) and had higher eGFR values: median (Q1; Q3) of 66 (42; 82) versus 39 (20; 69) mL/min/1.73 m^2^ (*p* < 0.001). Moreover, the diagnoses based on ICD-10 codes available in laboratory information system differed between the two groups (Table 2).

**Figure 5:**
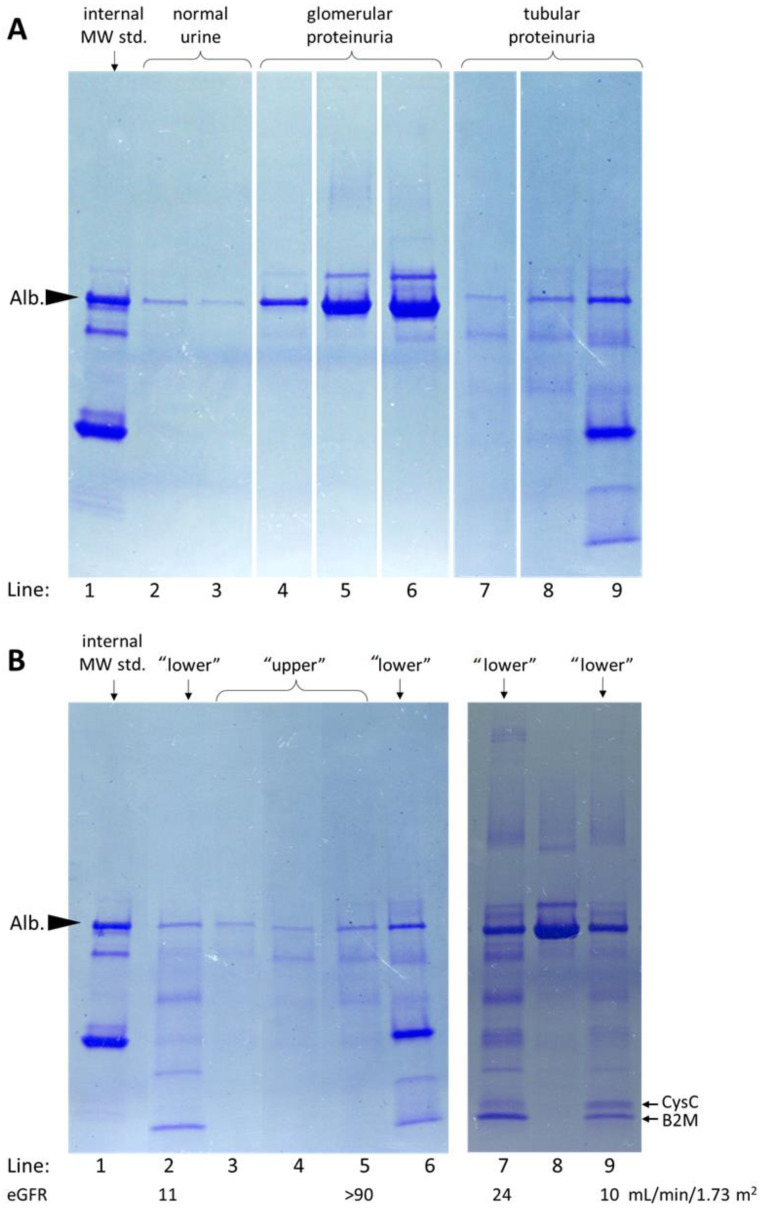
The representative SDS-PAGE separation patterns on 4–20% polyacrylamide gradient gels obtained in patients with proteinuria. The internal molecular weight standard (internal MW std.) is shown in line 1. Alb. indicates the albumin band. Panel (**A**): separation patterns characteristic for normal urine samples (lines 2 and 3), glomerular proteinuria of increasing grade 1 to 3 (lines 4–6) and tubular proteinuria (lines 7–9). Panel (**B**): the examples of “lower” tubular proteinuria in lines 2, 6, and 9; mixed glomerular—“lower” tubular proteinuria in line 7; and “upper” tubular proteinuria in lines 3, 4, and 5. Line 8 represents glomerular proteinuria. The values of eGFR are shown below the numbers of the respective lines. The arrows on the left indicate two low molecular weight protein fractions identified as containing cystatin C (CysC) and β_2_-microglobulin (B2M).

Figure 5 shows a representative examples of SDS-PAGE separations of a normal urine sample (a trace band of albumin) compared with the samples of tubular and glomerular proteinuria of varying severity: grade 1 characterized by an intense albumin band and a trace transferrin band, grade 2 by intense albumin and transferrin bands, and grade 3 by the additional presence of proteins of higher molecular mass. The presence of several fractions of low molecular weight proteins (i.e., below the molecular weight of albumin) characterized tubular proteinuria. Among the tubular patterns (including mixed tubular-glomerular proteinuria), we observed two distinct types, which we named the “upper” and the “lower” tubular proteinuria (Figure 5B). Moreover, we observed that the quantitative proportions of the selected protein fractions (identified as containing cystatin C and β_2_-microglobulin) varied among patients (Figure 5B, lines 7 and 9). The “upper” tubular proteinuria was characterized by the presence of proteins of molecular weight higher than 20 kDa and the absence of proteins with lower molecular weights. In contrast, the proteins with molecular weight below 20 kDa were present in samples of patients with “lower” tubular proteinuria. We observed that most (70%) patients with “upper” tubular proteinuria had eGFR values ≥60 mL/min/1.73 m^2^ while in most (87%) patients with “lower” tubular proteinuria, eGFR values were < 60 mL/min/1.73 m^2^ (Table 3). The hematologic lymphoid disorders were more common in patients with “upper” tubular proteinuria (59% versus 37%, *p* = 0.002) while renal disorders were two times less prevalent (25% versus 50%, *p* < 0.001).

**Figure 6:**
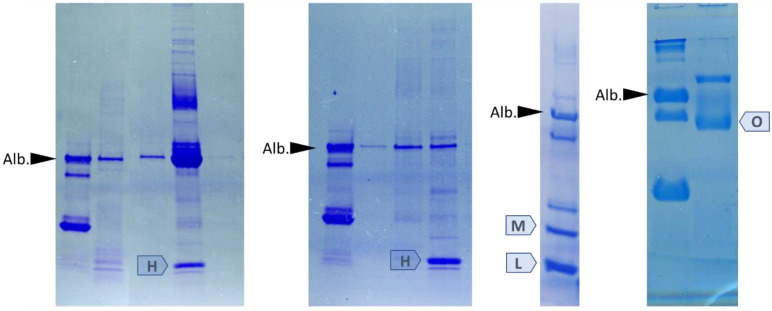
Examples of SDS-PAGE separation of urine samples containing hemoglobin (H), myoglobin (M), lysozyme (L), and ovalbumin (O). Alb. denotes albumin band.

In samples of patients with hemoglobinuria detected on urinalysis, we observed the presence of an intense low molecular weight fraction compatible with hemoglobin subunits (Figure 6). Moreover, the experience of our laboratory indicates that SDS-PAGE may help to explain rare causes of proteinuria, e.g., the presence of lysozyme or myoglobin in urine, or the addition of egg white to urine (Figure 6).

## 4. Discussion

As shown in our study, commercially available gradient polyacrylamide gels may be successfully used to evaluate proteinuria in patients. The standard method of Coomassie brilliant blue staining enables sufficient analytical sensitivity to detect clinically relevant concentrations of urinary proteins. The technique enables the separation of urinary proteins over the full spectrum of relevant molecular masses. The main albumin fraction is consistently visualized. In patient samples, protein fractions containing several “indicator” proteins may be easily distinguished. The pattern of the detected protein fractions enables the classification of the types of proteinuria (glomerular, tubular, and overload).

In our study, SDS-PAGE urine protein electrophoresis was performed on commercially available 4–20% gradient gels and self-casted 12.5% polyacrylamide gels. The use of gradient gels enabled the higher resolution of protein separation. Polyacrylamide gels with a constant acrylamide concentration allow for optimal separation of proteins in a limited molecular weight range while the gradient gels allow for the separation of proteins with a much wider range of molecular weights. Miller et al. [28] emphasized the advantages of gradient gels, such as the possibility of separating a mixture of proteins with a wide range of molecular weights (due to the decreasing diameter of the pores in the gel) and the high resolving capacity enabling the separation of mixture components, even with similar molecular weights. Previously, Lau and Woo [29] reported the use of gradient polyacrylamide gels for the assessment of proteinuria in 11 patients with various kidney diseases. They observed good agreement between protein band patterns and the results of kidney biopsies. Moreover, SDS-PAGE electrophoresis has been successfully used to diagnose renal diseases in veterinary medicine [30].

In our study, the limit of detection of albumin was 3 mg/L, which is clinically relevant and comparable to the sensitivity of the immunochemical methods. The method allows for semiquantitative assessment of protein concentrations in urine. The estimation of protein concentration using SDS-PAGE was reported by Maeda et al. [31] who determined the urinary concentrations of albumin and transferrin as early markers of chronic renal failure in cats. They used 10% polyacrylamide gels and observed visible bands of both albumin and transferrin at concentrations of 7.5 mg/L. Nonetheless, it must be acknowledged that Coomassie brilliant blue staining of different proteins is not uniform [32].

Additionally, based on electrophoretic separation of the same urine sample on different gels and at different times, we observed a good repeatability of SDS-PAGE. The repeatability of SDS-PAGE was previously studied by Scheller et al. [33]. In their study, relative standard deviations of molecular masses of the analyzed proteins (ovalbumin, carbonic anhydrase, bovine serum albumin, and phosphorylase B) were in the range of 1.5–1.7% [33].

The proteins on the polyacrylamide gel can be identified with different methods, e.g., using a calibration curve based on a molecular weight standard, using specific antibodies, or using mass spectrometry. In our study, the use of a commercially available standard allowed to estimate molecular weights corresponding to the relative migration distances of visible protein fractions. It has been shown that the determination of a protein molecular weight depends on the percentage of gel used for the electrophoretic separation and the type of the dye [34,35]. Of note, we observed differences in the electrophoretic mobility of the proteins present in the commercially available standard and the proteins present in urine. The albumin fraction in our electropherograms may be identified with high confidence by comparison with an internal standard and with other patient samples, as this fraction is consequently observed in nearly all urine samples (e.g., this is fraction 5 in the patient’s sample shown in Figure 4B,C). In our experiment, the molecular mass of albumin was underestimated based on the commercially available standard calibration curve. LC-MS/MS analysis confirmed the presence of albumin in fraction 5; the number of identified albumin peptides and the confidence of identification were the highest for this fraction.

The sensitive LC-MS/MS analysis of excised protein bands allowed us to detect several dozen proteins per fraction. However, the intensity of a band visible in our electropherograms reflects mainly the concentrations of the most abundant proteins. The LC-MS/MS analysis confirmed the presence of several “indicator” proteins in the analyzed urine sample [18,19,20,21,22]. These include the proteins known to be present in pathological urine in high concentrations. The structural fragments of immunoglobulin G were identified in fraction 1 (as numbered in Figure 4 and Table 1), and transferrin was identified in fraction 3; the proteins are indicative of glomerular proteinuria. The low molecular weight proteins indicative of tubular proteinuria included α1-microglobulin (fractions 8 and 9), retinol-binding protein (fraction 11–13), neutrophil gelatinase-associated lipocalin (fraction 12), cystatin C (fractions 14 and 15), and β2-microglobulin (fraction 15). Moreover, we detected immunoglobulin light chain (fractions 10 and 11), myoglobin (fraction 13), and lysozyme (fraction 14)—the proteins indicative for overload proteinuria. Uromodulin, cystatin C, and α_1_-microglobulin were also identified using specific antibodies on western blot analysis.

The identification of proteins in SDS-PAGE fractions is not straightforward. The lack of information on the composition of the commercially available standard and the possibility of proteolytic degradation of urinary proteins when passing through urinary tracts and during sample storage may result in differences in the electrophoretic mobility of urine proteins and the components of the standard. The methodological differences in the western blot include different percentages of polyacrylamide gels, different membranes (polyvinylidene difluoride—PVDF, nitrocellulose), and antibodies conjugated with different enzymes do not always allow to detect the same proteins with the method [36,37,38]. The identification of proteins using mass spectrometry also depends on the variant of SDS electrophoresis, type of staining, method of sample preparation, and its storage [39,40]. Nonetheless, the identification of individual proteins present in urine is not always necessary for diagnostic purposes. The characteristic patterns of fractions suggest the diagnosis of glomerular, tubular, or overflow proteinuria [29,30].

We analyzed 300 samples from patients with proteinuria and were able to reliably distinguish the main patterns of glomerular and tubular proteinuria. Moreover, the studied SDS-PAGE method enabled us to easily assess the severity of glomerular proteinuria: the selective (albumin and transferrin in urine) and non-selective (albumin, transferrin, and immunoglobulin in urine) glomerular proteinuria. In both tubular and glomerular proteinuria, the severity of proteinuria can be judged based on the intensity of the protein bands. Similarly, Suhail et al. conducted electrophoretic separations of urine samples from patients with acute renal failure and used their own multi-point scale to determine the intensity of tubular, glomerular, and mixed glomerulo-tubular proteinuria [24]. The analysis of ICD-10 codes retrieved from our laboratory information system revealed that nephrological diagnoses were most common in the studied patients with proteinuria, following by monoclonal gammopathies. The high proportion of patients with multiple myeloma and monoclonal gammopathy of undetermined significance in our study may be explained by the fact that the hospital serves as a reference center for the treatment of these diseases. In our analysis, multiple myeloma was more commonly associated with tubular proteinuria. Monoclonal gammopathies may result in a broad spectrum of renal injury; however, the cast nephropathy associated with tubular injury is most common [41]. It must be underlined that the tubular patterns we observed were associated with the presence of several low molecular weight proteins, not only the free light chains of immunoglobulins, although the monoclonal FLCs were present in some of the patients (the representative examples are shown in Figure 5). Moreover, we only had access to the ICD-10 codes of the main diagnoses, not knowing the phase of disease and the stage of treatment.

We observed two types of tubular or mixed proteinuria, which were related to GFR values observed in patients and the diagnoses. “Upper” type of tubular proteinuria was characterized by the presence of proteins with molecular weights ≥ 20 kDa, the GFR value remaining at the level ensuring efficient removal of uremic toxins (>60 mL/min/1.73 m^2^ in most patients) and the higher prevalence of multiple myeloma. “Lower” tubular proteinuria was characterized by the presence of proteins with molecular weight < 20 kDa and a GFR value < 60 mL/min/1.73 m^2^ (down to 10 mL/min/1.73 m^2^) in most patients; renal diagnoses including CKD were more common in these patients. A possible mechanism of “upper” type of tubular proteinuria is a failure of the renal tubular resorption mechanisms in line with maintained efficient glomerular filtration. On the other hand, “lower” tubular proteinuria may occur in the case of failure of the glomerular filtration, which leads to the accumulation of proteins with molecular weights <20 kDa in blood. Physiologically, such proteins are freely filtered in the glomeruli into urine. “Lower” tubular proteinuria can also be caused by co-occurring failure of glomerular filtration and tubular resorption. The increase in the concentrations of low molecular weight proteins, such as retinol binding protein, β-trace protein, cystatin C, and β_2_-microglobulin in both serum and urine of patients with CKD in correlation with a decrease in GFR was described by Donadio [42]. In samples with “lower” tubular proteinuria, we observed evident differences in the proportions of individual protein fractions, especially those containing 11–14 kDa proteins (identified as cystatin C and β2-microglobulin). This may be related to different mechanisms of their reabsorption within the renal tubules [43,44]. Inconsistent literature data show that protein reabsorption within the tubules may be caused by selective mechanisms specific to a given protein. The difference in the proportion of individual urine proteins may therefore indicate distinct mechanisms of tubular damage [45].

The studied SDS-PAGE method allows the detection of proteins related to overload proteinuria, including FLC, hemoglobin subunits, myoglobin, and lysozyme. Urine protein electrophoresis is recommended by the International Myeloma Working Group for the diagnosis and management of myeloma renal impairment [46]. SDS electrophoresis is an excellent tool for the detection of Bence–Jones protein in the urine of multiple myeloma patients. Immunoglobulin light chains in SDS-PAGE performed in non-reduced conditions appear in the forms of a dimer (46 kDa) and a monomer (23 kDa), resulting in a characteristic electrophoretic pattern. The presence of additional proteins such as albumin, low or high molecular weight proteins clearly indicate kidney structures damaged as a result of filtration and reabsorption of an excess of nephrotoxic monoclonal immunoglobulin [47,48].

Hemoglobin in urine may be associated with both bleeding in the urinary tract and intravascular hemolysis, while erythrocyturia may also be a symptom of glomerulopathy [49]. Myoglobin is released into the circulation as a result of skeletal muscle damage or a heart attack, may be present in urine in high concentrations, and is highly nephrotoxic. The quantitative immunochemical methods used for the assessment of myoglobinuria may give discrepant results, which has been attributed to the instability of myoglobin in stored urine [50]. Nonetheless, even a partially degraded myoglobin will still be visible on an electropherogram. The presence of lysozyme in serum and urine is observed in patients with monocytic and monomyelocytic leukemia [51].

Finally, the use of SDS-PAGE may reveal the rare causes of proteinuria. In our practice, we encountered a patient who deliberately added an egg white to urine, leading to the extended process of the differential diagnosis of proteinuria. The use of SDS electrophoresis allowed the identification of ovalbumin in the patient’s urine.

Several electrophoretic techniques have been used to separate urine proteins. Historically, urine specimens concentrated via either the removal of water or centrifugation were electrophoretically separated on cellulose acetate and later on agarose and high-resolution agarose gels. Currently, capillary electrophoresis of urinary proteins and mass spectrometry are increasingly used in scientific studies concerning kidney diseases [52,53,54]. The electrophoretic profile of urine proteins is a complex image of many fractions. The interpretation of the results requires experience from the laboratory staff. In modern profit-optimized laboratories with constantly rotating staff, acquiring sufficient skills may be problematic. Protein profiles associated with various types of proteinuria (e.g., glomerular and tubular) may overlap, making the interpretations of results complicated. It must be ensured that different team members describe the same profiles in the same way, since a written report is the sole way of communication between laboratories and clinicians. An even bigger problem is the harmonization of results between laboratories [55,56,57]. Nonetheless, the clinical usefulness of urinary protein electrophoresis has been admitted in recent clinical practice guidelines, e.g., regarding or glomerular diseases [56] or renal impairment associated with myeloma [46]. In both ours and others [29] experience, SDS-PAGE is a suitable method for urinary protein analysis. The manual workload associated with SDS-PAGE may be minimized by the use of the commercially available gels. Moreover, in our experience, the studied method allows for repeatable and fairly easy interpretation of protein fraction patterns, suggesting the main types of proteinuria in most cases.

## 5. Conclusions

Our study has several limitations. We could not compare the studied method with a gold standard, because there is no gold standard for the determination of the type of proteinuria. Moreover, our analysis of the association between the type of proteinuria and the clinical diagnosis must be considered provisional. Our study concentrated on the method characterization and was laboratory based. We only had an access to the main ICD-10 codes available in our laboratory information system, which poorly characterize the patient conditions and are often irrelevant or missing. Therefore, the association between SDS-PAGE urinary protein patterns and the clinical conditions deserve more insight in well-planned clinical studies.

Our study shows that SDS-PAGE separation of urine proteins on commercially available 4–20% gradient gels is a reliable technique to diagnose proteinuria and differentiate its main clinically relevant types (glomerular, tubular, and overflow). The technique may be useful in the differential diagnosis of patients with proteinuria of unknown causes. The method may be successfully used in clinical practice in both human and veterinary medicine as well as in research.

## Figures and Tables

**Table 1:** LC-MS/MS identification of the main proteins in fractions obtained using SDS-PAGE separation of urine proteins on 4–20% gradient gels. The protein fractions are numbered as shown in Figure 4B.

Protein Fraction Number	Protein Identification		
	Protein	PSMs	Molecular Weight [kDa]
1 ^a^	1. Ceruloplasmin 2. Complement factor H	28 17	122.13 139.01
2	1. Plasminogen 2. Complement component C6 3. Complement component C7 4. Inter- α-trypsin inhibitor heavy chain H	29 17 12 11	90.51 104.72 93.46 103.29
3	1. Serotransferrin ^b^ 2. Coagulation factor XIII B chain 3. Kininogen 4. Afamin 5. Protein disulfide-isomerase A4	550 104 65 34 18	77.01 75.46 71.91 69.02 72.89
4	1. Albumin 2. Complement component C9	1073 55	69.23 63.13
5	1. Albumin 2. Hemopexin 3. Antithrombin-III	2756 308 104	69.23 51.64 52.57
6	1. Albumin 2. Pigment epithelium-derived factor 3. Vitamin D-binding protein 4. Hemopexin 5. Apolipoprotein A-IV 6. Antithrombin-III	1135 411 295 147 140 135	69.23 46.28 52.88 51.64 45.34 52.57
7	1. Apolipoprotein A-IV 2. Pigment epithelium-derived factor 3. Vitamin D-binding protein 4. EGF-containing fibulin-like extracellular matrix protein 1	471 163 124 22	45.34 46.28 52.88 54.60
8	1. Protein AMBP ^c^ 2. Zinc-alpha-2-glycoprotein 3. β_2_-glycoprotein 4. Insulin-like growth factor-binding protein 2	276 62 51 22	38.97 34.24 38.27 34.79
9	1. Protein AMBP ^c^ 2. CD-5 antigen like 3. Mimecan 4. Apolipoprotein A-I 5. Corticotropin-releasing factor-binding protein	933 42 31 29 28	38.97 38.06 33.90 30.76 36.12
10	1. Complement factor D 2. Apolipoprotein A-I 3. Immunoglobulin κ light chain 4. Phosphatidylethanolamine-binding protein 4 5. Prostaglandin-H2 D-isomerase	532 438 280 130 87	27.02 30.76 23.36 25.72 21.02
11	1. Immunoglobulin κ light chain 2. Retinol-binding protein 4 3. Apolipoprotein A-I 4. Neutrophil gelatinase-associated lipocalin	499 132 58 56	23.36 22.99 30.76 22.57
12	1. Retinol-binding protein 4 2. Protein FAM3C 3. Tetranectin 4. Neutrophil gelatinase-associated lipocalin 5. Transgelin 6. Ganglioside GM2 activator 7. Metalloproteinase inhibitor 2	777 56 27 19 18 16 16	22.99 24.67 22.52 22.57 22.60 20.83 24.38
13	1. Retinol-binding protein 4 2. Retinoic acid receptor responder protein 2 3. Myoglobin 4. Peptidyl-prolyl cis-trans isomerase A 5. Transthyretin	234 96 82 52 33	22.99 18.61 17.17 18.00 15.88
14	1. Cystatin C 2. Lysozyme C 3. Fatty acid-binding protein, adipocyte 4. Proflin-1 5. Fatty acid-binding protein, liver 6. Hemoglobin subunit β	608 288 107 51 38 27	15.79 16.53 14.71 15.05 14.20 15.99
15	1. β_2_-microglobulin 2. Cystatin C 3. Cystatin M	251 82 34	13.71 15.79 16.50

^a^—notably, in fraction 1, immunoglobulin γ heavy chains, κ light chains and constant fragments were identified with high confidence (Supplementary File 2); ^b^—also known as transferrin; ^c^—a precursor protein for α1-microglobulin. AMBP, α-1-microglobulin/bikunin precursor; CD, cluster of differentiation; FAM3C, family with sequence similarity 3; PSM, peptide sequence match.

**Table 2:** Diagnoses reported in the laboratory information system in association with 300 urine samples from patients with proteinuria. The samples were separated using 4–20% gradient polyacrylamide gels. A tubular or mixed tubular-glomerular pattern was observed in 204 samples and a pure glomerular pattern in 96 samples.

ICD-10 Code	Name of Disorder	All Samples, *n* (%)	Tubular/Mixed Pattern, *n* (%)	Pure Glomerular Pattern, *n* (%)	*p*-Value
Hematologic lymphoid disorders:		117 (39)	91 (45)	26 (27)	0.004
C90.0	Multiple myeloma	112 (37)	87 (43)	25 (26)	0.006
D47.2	MGUS	4 (1)	3 (1)	1 (1)	1.0
C82.9	Follicular lymphoma	1 (0.3)	1 (0.5)	0	1.0
Renal disorders:		133 (44)	83 (41)	50 (52)	0.064
N18.x	Chronic kidney disease	59 (20)	37 (18)	22 (23)	0.3
Z94.0	Kidney transplant status	18 (6)	12 (6)	6 (6)	0.9
N03.x-N06.x	Glomerular diseases	36 (12)	20 (10)	16 (17)	0.087
N17.x	Acute kidney injury	9 (3)	7 (3)	2 (2)	0.7
N19.x	Unspecified kidney failure	6 (2)	2 (1)	4 (4)	0.085
E10.2, E11.2	Type 1/type 2 diabetes mellitus with renal complications	3 (1)	3 (1)	0	0.6
N28.x	Other disorders of the kidney and ureter	2 (0.7)	2 (1)	0	1.0
Other potentially relevant disorders:		16 (5)	9 (4)	7 (7)	0.3
M31.3	Wegener granulomatosis	2 (0.7)	0	2 (2)	0.1
M35.0	Sjögren syndrome	1 (0.3)	1 (0.5)	0	1.0
Cx (other than C90.x)	Malignant neoplasms	9 (3)	2 (1)	7 (7)	0.006
Ix	Diseases of the circulatory system	5 (2)	5 (2)	0	1.0
Uninformative, irrelevant, or missing codes:		33 (11)	22 (11)	11 (11)	0.9

ICD-10, International Statistical Classification of Diseases and Related Health Problems 10th Revision; MGUS, Monoclonal gammopathy of undetermined significance.

**Table 3:** Estimated glomerular filtration rate (eGFR) values observed in patients with “upper” and “lower” tubular proteinuria. The eGFR values were compared between the two groups of patients using the Mann–Whitney test.

Type of Tubular Proteinuria	Number of Samples	eGFR Value <60 mL/min/1.73 m^2^, n (%)	Median (Q1; Q3) eGFR, mL/min/1.73 m^2^	*p*-Value
“upper”	73	22 (30)	71 (55; 90)	<0.001
“lower”	131	114 (87)	31 (13; 39)	

Q1–lower quartile; Q3–upper quartile; n–number of patients; gGFR–estimated glomerular filtration rate.

## Data Availability

The data presented in this study are available on request from the corresponding author.

## Supplementary Materials

The following supporting information can be downloaded at: https://www.mdpi.com/article/10.3390/diagnostics13091513/s1: Supplementary File 1 containing the details of preparing self-cast polyacrylamide gels; Supplementary File 2 containing the full list of proteins identified using LC-MS/MS in the 15 analyzed protein fractions; Supplementary Figure S1 presenting the western blot detection of uromodulin, α1-microglobulin, and cystatin C in urine samples.
