# Supplementary material for: SDS Electrophoresis on Gradient Polyacrylamide Gels as a Semiquantitative Tool for the Evaluation of Proteinuria
Source: Diagnostics (Basel). 2023 Apr 23;13(9):1513. doi: 10.3390/diagnostics13091513 (PMC10177418; doi:10.3390/diagnostics13091513)
Supplement: Supplementary file 1 [file diagnostics-13-01513-s001.zip › SDS-PAGE_proteinuria_Supplementary_Figure_S1.pdf]

# SDS electrophoresis on gradient polyacrylamide gels as a semiquantitative tool for the evaluation of proteinuria

Paulina Mazur, Paulina Dumnicka, Joanna Tisończyk, Anna Ząbek-Adamska, and Ryszard Drożdż

## Supplementary Figure S1

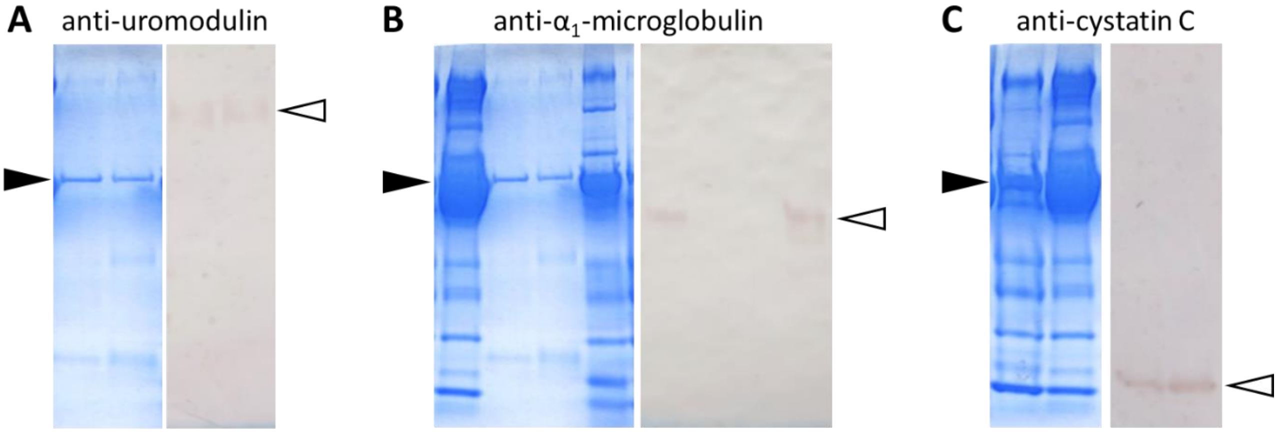


**Supplementary Figure S1.** Urine samples from patients with proteinuria separated on 4-20% polyacrylamide gradient gels (left panels) and the respective nitrocellulose membranes with transferred urine proteins visualized using anti-uromodulin (A), anti $\alpha$1-microglobulin (B) and anti-cystatin C antibodies. The black arrowheads indicate the albumin bands on polyacrylamide gels; the open arrowheads indicate the respective proteins' bands on nitrocellulose membranes.
