# Supplementary material for: SDS Electrophoresis on Gradient Polyacrylamide Gels as a Semiquantitative Tool for the Evaluation of Proteinuria
Source: Diagnostics (Basel). 2023 Apr 23;13(9):1513. doi: 10.3390/diagnostics13091513 (PMC10177418; doi:10.3390/diagnostics13091513)
Supplement: Supplementary file 1 [file diagnostics-13-01513-s001.zip › SDS-PAGE_proteinuria_Supplementary_file_1.pdf]

# SDS electrophoresis on gradient polyacrylamide gels as a semiquantitative tool for the evaluation of proteinuria

Paulina Mazur, Paulina Dumnicka, Joanna Tisończyk, Anna Ząbek-Adamska, and Ryszard Drożdż

## Supplementary file 1

### Self-made polyacrylamide gels

*Separating buffer*

Separating buffer was prepared by dissolve 18.17 g of Trizma base and 0.4 g of SDS in 100 mL of distilled water. pH was adjusted to 8.8 with hydrochloric acid.

*Concentrating buffer*

Concentrating buffer was prepared by dissolve 6.05 g of Trizma base and 0.4 g of SDS in 100 mL of distilled water. pH was adjusted to 6.8 with hydrochloric acid.

*Gel casting*

Polyacrylamide gels were prepared in a discontinuous system: a layer of a concentrating gel was poured over the layer of a separating gel. Depending on the required gel percentage, appropriate volumes of the solutions were added (Supplementary table 1), starting with distilled water, then 30% acrylamide, separating buffer (pH 8.8, 1.5 M tris-HCl) and TEMED. Finally, directly before pouring the gel between the previously prepared glass plates, gel polymerization initiator – 10% ammonium persulfate (APS), was added to the solution. 0.5 mL of butanol was applied on the separating gel to ensure correct polymerization process. The separating gel was polymerized for about 20 minutes, after which the butanol layer was removed, and a layer of concentrating gel was poured over the separating gel. The concentrating gel was prepared by mixing the appropriate volumes (Supplementary table 1) of distilled water, 30% acrylamide, concentrating buffer (pH 6.8, 0.5 M tris-HCl), TEMED and finally 10% APS. The prepared solution was poured onto the polymerized separating gel. Then, a comb of 1.5 mm thickness was placed between the glass plates. After the concentrating gel was polymerized, the prepared gel was placed in a moist chamber and stored at 4°C. The comb was removed just before electrophoresis process.

**Supplementary table 1.** Composition of self-made acrylamide gels

| Constituent | Separating gel (12.5% acrylamide) | Concentrating gel (4% acrylamide) |
|---|---|---|
| 30% acrylamide, mL | 4.2 | 0.7 |
| Separating buffer, pH 8.8, mL | 2.5 | - |
| Concentrating buffer, pH 6.8, mL | - | 1.25 |
| 10% ammonium persulphate, mL | 0.05 | 0.05 |
| Distilled water, mL | 3.4 | 3.1 |
| TEMED, μL | 5 | 5 |

### Sample buffer

Sample buffer was prepared by mixing 4 mL of 0.1% bromophenol blue solution, 4 mL of 40% sucrose solution and 8 mL of 20% SDS solution.

### Electrode buffer

Electrode buffer was prepared by dissolve 30 g of Trizma base, 10 g of SDS and 144 g of aminoacetic acid in 1 L of distilled water; pH was adjusted to 8.3 with hydrochloric acid.
